# Molecular and Physiological Perspectives of Abscisic Acid Mediated Drought Adjustment Strategies

**DOI:** 10.3390/plants10122769

**Published:** 2021-12-15

**Authors:** Abhilasha Abhilasha, Swarup Roy Choudhury

**Affiliations:** Department of Biology, Indian Institute of Science Education and Research (IISER) Tirupati, Tirupati 517507, Andhra Pradesh, India; abhilashaphd@students.iisertirupati.ac.in

**Keywords:** ABA, cuticle, dormancy, drought, senescence, signaling, stomata

## Abstract

Drought is the most prevalent unfavorable condition that impairs plant growth and development by altering morphological, physiological, and biochemical functions, thereby impeding plant biomass production. To survive the adverse effects, water limiting condition triggers a sophisticated adjustment mechanism orchestrated mainly by hormones that directly protect plants via the stimulation of several signaling cascades. Predominantly, water deficit signals cause the increase in the level of endogenous ABA, which elicits signaling pathways involving transcription factors that enhance resistance mechanisms to combat drought-stimulated damage in plants. These responses mainly include stomatal closure, seed dormancy, cuticular wax deposition, leaf senescence, and alteration of the shoot and root growth. Unraveling how plants adjust to drought could provide valuable information, and a comprehensive understanding of the resistance mechanisms will help researchers design ways to improve crop performance under water limiting conditions. This review deals with the past and recent updates of ABA-mediated molecular mechanisms that plants can implement to cope with the challenges of drought stress.

## 1. Introduction

As sessile organisms, plants are exposed to an ever-changing environment during their entire lifespan. Plants have acquired adaptive physiological and molecular mechanisms by diverse mitigating strategies in the course of evolution to cope with unfavorable conditions. Predominantly, plants are exposed to both abiotic and biotic stress at various stages of their development. Biotic stress includes the attack by pests and pathogens, e.g., bacteria, viruses, fungi, and nematodes, whereas abiotic stress includes drought, flooding, temperature, salinity, nutrient deficiency, heavy metals, and ultraviolet radiation [1,2]. Consequently, they adversely affect the productivity of the crop, which is a major obstacle to attaining global food security essential for the continuously growing world population. Among the abiotic stresses, drought has become a major plague as a result of climate-change scenarios around the world and a certain percentage of developing countries will face water scarcities by 2030 (FAO 2003) [3]. In the least developed countries (LDCs) and low to middle-income countries (LMICs), over 34% of crop and livestock production was reduced by drought from 2008-2018 (FAO 2021) [4]. Among the world’s major crops, rice (>50% yield reduction) was more sensitive towards drought compared to maize (39.3% yield reduction) and wheat (20.6% yield reduction) under comparable water reduction (approximately 40%) [5,6]. Under drought severity growing from moderate to exceptional, the yield loss risk is anticipated to increase by 9%–12%, 5.6%–6.3%, and 18.1%–19.4% in wheat, maize, and rice, respectively [7].

Water scarcity in the soil directly constrains physiological functions in plants, such as leaf growth, photosynthetic capacity, nutrient uptake, stomatal conductance etc. [8]. The limited water availability initiates when the transpiration rate is higher than the amount of water absorbed by plant roots [9]. Typically, plants need water from seedling to the reproductive stage to transport nutrients, for photosynthesis, and to maintain the turgidity of cell walls and various cellular processes [10]. Not having adequate water poses a severe threat to seed germination and seedling growth in plants with decreasing osmotic potential [11,12,13,14,15,16]. Prolonged drought condition reduces uptake of nutrients by root, leaf water potential, transpiration rate, water-use efficiency and stomatal conductance essential for both vegetative and reproductive growth [11]. Under drought, the interruption of water flow between xylem and adjacent cells negatively affects turgor pressure, which in turn decreases cell elongation, consequently resulting in reduced plant height, leaf area, and crop yield [17]. A decline in stomatal conductance is responsible for a net reduction in photosynthesis due to inhibited CO_2_ assimilation [18]. These physiological changes lead to the production of reactive oxygen species (ROS), which promotes oxidative stress via the impairment of cell membranes, nucleic acids, and proteins to destabilize cellular functions [19,20]. To abate the effects of drought stress, plants respond in very complex ways, bringing morphological and physiological adaptation. Such processes include closing stomata, increased root growth, decreased stem and leaf expansion, cuticular wax biosynthesis, and shortening life cycle [17,21]. In addition, plants have evolved efficient antioxidant machinery, including enzymatic and non-enzymatic systems to attenuate the effect of ROS [19]. The promoter of catalase gene *Cat2* in wheat contains ARE (antioxidant responsive element), which is induced by H_2_O_2_ during drought [22]. Besides, plants also regulate osmotic adjustment via the accumulation of soluble sugars, free amino acid, and proline required for normal cellular homeostasis [23]. To counter drought stress by triggering different mitigating strategies is directly controlled by a well synchronization of gene expression, such as *RD22, RD29B, RD20A, Gly* (glyoxalase I family) etc., predominantly modulated by multiple hormonal signaling [24].

Along with many fundamental processes plant hormone abscisic acid (ABA), a key regulator of abiotic stress resistance, plays an important role in mediating drought stress responses in coordination with other plant hormones. Drought stress enhances cellular calcium level, which leads to a calcium-dependent phosphorylation cascade to activate the essential genes required for ABA biosynthesis, such as zeaxanthin oxidase (ZEP), 9-cis-epoxycarotenoid dioxygenase (NCED), ABA-aldehyde oxidase (AAO), and molybdenum cofactor sulphurase (MCSU) [25]. Synthesized active ABA is predominantly accumulated in the vascular tissue of leaves and transported to sites of action for stress response [26,27]. ABA is also stored in vacuole in biologically inactive form by conjugated with glucose ester (ABA-GE) [28]. In addition to the core biosynthetic pathway, endogenous ABA levels increase in response to drought stress through the hydrolyzation of an inactive form of ABA, ABA-GE, to active form [29]. The enhancement of active ABA concentration can cause both repression and increased expression of ABA-responsive genes via ABA signaling machinery.

Forward genetics screening of pyrabactin resistant mutants has identified pyrabactin resistance 1 (PYR1)/PYR1-Like (PYL)/regulatory component of ABA receptor (RCAR) genes as ABA receptors [30,31,32]. There are two major steps to ABA signaling that include PYR1/PYL/RCAR receptor activation by ABA to negatively regulate PP2Cs (Protein phosphatase 2C) and concurrent activation of SNF1-related protein kinase 2 (SnRK2s) to modulate downstream genetic circuits. PYR/PYL/RCAR receptors are homologous to the START (steroidogenic acute regulatory) domain superfamily, which contains a conserved helix-grip motif to generate a central hydrophobic ligand-binding pocket essential for lipids and hormones binding [33]. After binding to the central hydrophobic ligand-binding pocket, ABA creates conformational change in the receptor by forming a gate-latch interface by closing the gate. This structural alteration facilitates binding ABA bound receptor to the PP2C active site to further lock the receptor, thereby forming a receptor-ABA-PP2C complex via gate-latch-lock mechanism [34,35].

Among the other plant phosphatase, PP2Cs, an evolutionarily conserved serine (Ser)/threonine (Thr)-specific phosphatases (STPs) or metal-dependent protein phosphatases (PPMs), act as major negative regulators of ABA signal transduction pathways. The genetic screen for ABA-insensitive mutants and sequence similarity identified multiple PP2Cs in Arabidopsis, such as ABA-INSENSTIVE1 (ABI1), ABI2, ABA-HYPERSENSITIVE GERMINATION1 (AHG1), AHG3/AtPP2CA, HOMOLOGY TO ABI1 (HAB1), and HAB2 [36,37,38,39,40]. PP2Cs are Mg^2+^/Mn^2+^–dependent monomeric enzymes, which predominantly inhibit activation of the ABA-responsive transcription factors (TFs) by dephosphorylation of SnRK2s. When endogenous ABA levels are upregulated by developmental or environmental cues, ABA bound PYR/PYL/RCAR receptors interact with PP2Cs to inhibit its protein phosphatase activity, resulting in the release of active SnRK2s [34,41,42,43].

Protein phosphorylation of Snf1-Related Kinases2 (SnRK2s) is one of the major cellular events in ABA signaling. Among the three subclasses: I, II, and III, Subclass III SnRK2s act as positive regulators of ABA signaling as they are rapidly activated by ABA [44]. In Arabidopsis, three members of Subclass III SnRK2s (SRK2D/SnRK2.2, SRK2I/SnRK2.3, and SRK2E/OST1/SnRK2.6) are activated by ABA within 30 min. Generally, SnRK2s are plant-specific Ser/Thr protein kinases, either auto-phosphorylated or trans-phosphorylated by other kinases when ABA receptors specifically sequester PP2Cs, thereby facilitating SnRK2s activation. The active SnRK2s by their well-conserved kinase catalytic domain positively regulate downstream ABA-responsive genes via the phosphorylation of TFs, which include bZIP transcription factors like ABRE (ABA-responsive element)-binding (AREB) proteins or ABRE-binding factors (ABFs) [45,46,47]. SnRK2s mediated AREB/ABFs phosphorylation is a crucial ABA-dependent regulation. These phosphorylated AREBs or ABFs bind to conserved ABA-responsive elements (ABRE) present on the promoter of ABA-regulated genes to up-regulate several downstream genes, for example, *RD29B* [24]. In addition, several other TFs, including MYC (myelocytomatosis), MYB (myeloblastosis), DREB2 (drought-responsive element binding), NAC (NAM, ATAF1,2, and CUC), AP2/ERF (apetala 2/ethylene responsive factor), basic leucine zipper, and HD-ZIP (homeodomain leucine zipper), greatly influence plant abiotic stress resistance via ABA-dependent and ABA-independent signal transduction pathways by binding with specific cis-acting elements present in promoter regions of several stress-induced genes [48,49,50]. For instance, the *RD29* (response to dessication) genes are regulated by AREBs and DREBs TFs via both ABA-dependent and independent signal transduction pathways [51]. MYC (myelocytomatosis) and MYB (myeloblastosis) family’s TFs are involved in the ABA-dependent pathway for the up-regulation of abiotic stress-responsive genes like *RD22* [52,53], which is involved in drought stress response via stomatal regulation [54]. In contrast, DREB proteins that bind to DRE cis-elements induce an ABA-independent stress-responsive gene expression, leading to stress resistance ability via the accumulation of osmoprotectants like proline, sucrose [34,55,56]. For example, DREB triggers the expression of *RD29A* gene during drought stress without involvement of ABA [56,57]. In addition, heterotrimeric G-proteins regulate the ABA signaling pathway and drought resistance by manipulating downstream signaling cascades [58,59,60]. For example, the two subunits of G-protein in rice, *qPE9-1* (Gγ subunit) and *RGB1* (Gβ subunit), show contrast regulation of ABA dependent stress responses acting as negative and positive regulators, respectively [61].

## 2. General Aspects of Plant Drought Stress

ABA is the major phytohormone that accumulates in the presence of drought stress to modulate an array of biochemical and physiological changes for acclimatization against stress conditions via ABA-mediated a wide variety of gene expression [62]. Various morpho-physiological alterations were induced by ABA during short-term and prolonged exposure of plants in drought [63]. Here, this review deals with the molecular adaptive mechanisms that plants can implement to combat various drought stress challenges.

### 2.1. Stomata Closure

Stomata are specialized structures constituting a pair of guard cells enclosing a central aperture through which CO_2_ gas enters the leaf interior for photosynthesis and concomitant loss of water vapor by transpiration [64]. Under water-deficit conditions, plants can decrease the stomatal pore size to control gas exchange and transpiration rates via reducing guard cell turgor pressure and stomatal conductance [65,66]. Predominantly, enhanced ABA synthesis in guard cells permits stomatal closure under drought. Besides enzymatic activation of ABA biosynthesis enzymes, an inert ABA conjugate activation by β-glucosidase is also responsible for ABA accumulation in leaf guard cells [67,68].

**Figure 1:**
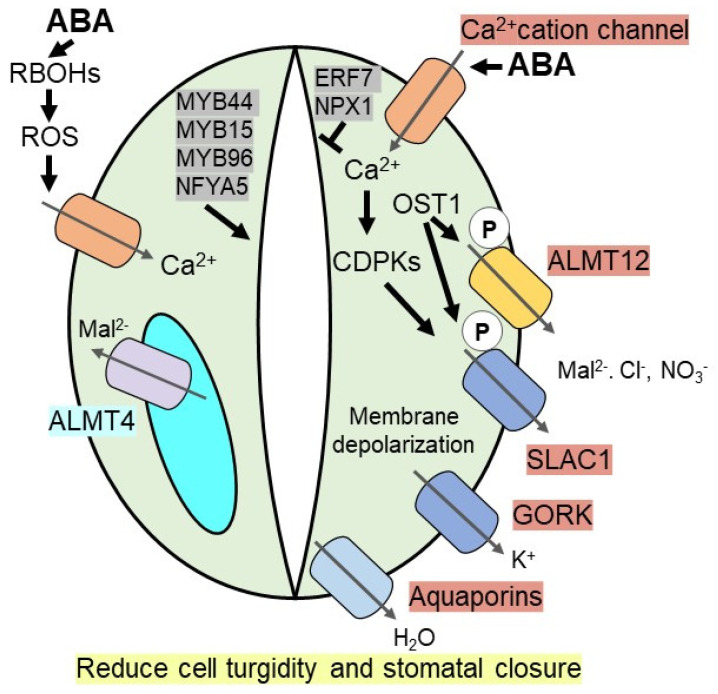
**ABA induces stomatal closure under drought stress.** During drought, accumulated ABA inside guard cells induces Ca^2+^ level, which activates CDPKs (calcium-dependent protein kinases), thereby triggering SLAC1 (slow anion channel-associated 1) channel. Ca^2+^ independent kinase, OST1 (open stomata 1), phosphorylates SLAC1 anion channel as well as R-type ALMT12 (aluminium-activated malate transporter 12) anion channel to efflux the anions such as malate (Mal^2−^), Cl^−^ and NO^3−^. It promotes plasma membrane depolarization to efflux K^+^ ion via GORK (guard cell outwardly rectifying K^+^ channel) and water via aquaporins. ABA induces RBOHs (NADPH oxidase/respiratory burst oxidase homolog) on guard cells membrane to generate ROS (reactive oxygen species), which promotes Ca^2+^ level. A vacuolar anion channel, ALMT4 triggers Mal^2−^ ion outside of vacuole required for stomatal closure. Several transcription factors [MYB44, MYB15, MYB96, NFYA5 (nuclear transcription factor Y subunit A-5), ERF7 (ethylene responsive factor), NPX1 (Nuclear Protein X1)] either positively or negatively promote ABA induced stomatal closure to enhance drought resistance.

Elevated ABA in guard cells under drought stress induces the level of calcium in the cytosol by triggering Ca^2+^ influx via non-selective Ca^2+^ cation channels or hyperpolarization-activated Ca^2+^ channels. Increased levels of cytosolic calcium activate calcium-dependent protein kinases (CDPKs), which induces SLAC1 (Slow Anion Channel-Associated 1), a S-type anion channel, via phosphorylation [69,70]. For example, activation of SLAC1 is attenuated in CDPK mutants (*cpk3cpk6*) [71]. In addition, OST1 (open stomata 1), a calcium-independent SnRK2-type kinase, phosphorylate SLAC1 and also R-type anion channels, like aluminium-activated malate transporter 12/quickly activating anion channel 1 (ALMT12/QUAC1) in Arabidopsis [65,72,73]. Phosphorylated anion channels efflux anions (malate (Mal^2−^)_,_ Cl^−^ and NO^3−^) out of the guard cells, which subsequently promote plasma membrane depolarization to drive K^+^ efflux through the voltage-dependent outward K^+^ (K^+^_out_) channel like GORK (guard cell outwardly rectifying K^+^ channel). The constant efflux of ions from the guard cells promotes the efflux of water out of the cell via aquaporins to reduce cell turgidity, thereby facilitating stomatal closure [28,64,65,67] (Figure 1).

Apart from plasma membrane-bound anion channels, the ABA-mediated unknown dephosphorylation mechanism activates vacuolar anion channels, subsequently inducing stomatal closure or impeding stomatal opening [74]. Recently discovered ALMT4 (aluminum activated malate transporter 4), a vacuolar anion channel in Arabidopsis guard cells, is essential for ABA-mediated stomatal closure as knockout mutants of *almt4* are unable to control stomatal movement in response to ABA or drought stress. During drought, stress-induced ABA-mediated stomatal closure, ALMT4 is involved in malate (Mal^2−^) efflux from the vacuole [75]. In addition, ABA induces two NADPH oxidases, *AtrbohD* and *AtrbohF,* to generate reactive oxygen species (ROS), like oxygen radicals and H_2_O_2_, which act as a positive regulator for stomatal closure by increasing influx of Ca^2+^ through the Ca^2+^ channel [76,77]. Increased cytosolic Ca^2+^ governs multiple Ca^2+^ dependent kinases to regulate ion channels as well as ROS producing enzymes such as RBOH (NADPH oxidase/respiratory burst oxidase homolog) required for stomatal closure (Figure 1).

Transcriptional regulation plays an essential role in ABA-mediated stomatal closure. Recent studies show that several R2R3 MYB TFs are involved in the modulation of guard cells in the ABA-dependent pathway. For instance, in Arabidopsis both *AtMYB44* and *AtMYB15* overexpression lines are more sensitive to ABA-induced stomatal closure compared to wild-type plants. Therefore, these transgenic lines exhibit remarkably improved resistance to drought stress [78,79]. During water deficit conditions, contrasting stomatal aperture is observed in MYB96 overexpressing and knockout mutant plants. Overexpression of *MYB96* results in increased resistance to drought stress as it triggers stomatal closure, whereas its knockout mutants show a lesser extent of decrease in stomatal aperture under drought, indicating that MYB96 plays a role in controlling stomatal opening [80]. Besides MYB TFs, several other ABA inducible TFs regulate stomatal movement. For example, AtERF7, an APETALA2/ethylene-responsive element-binding protein (AP2/EREBP) family of TFs, acts as a negative regulator of stomatal closure. Thus, *aterf7* RNAi lines show increased ABA sensitivity and enhanced survival compared to wild-type in Arabidopsis [81]. NFYA5 (Nuclear transcription factor Y subunit A-5) TF belongs to the Arabidopsis NF-YA family and is critically important in stomatal movement. Overexpression of *NFYA5* significantly enhances stomatal closure and increases plant survival under drought stress by positively regulating other drought-responsive genes via binding at CCAAT box cis-element [82]. NPX1 (Nuclear Protein X1) TF represses genes involved in ABA synthesis and ABA signaling; thus, *npx1* null mutant shows higher ABA-induced stomatal closure and water deficit resistance than wild-type [83] (Figure 1). In rice, ABA inducible SNAC1 (STRESS RESPONSIVE NAC1) promotes ABA-induced stomatal closure and enhanced drought resistance [84,85]. Apart from TFs, E3 Ub ligase genes in Arabidopsis, such as *AtPUB18* and *AtPUB19,* are negative regulators of ABA mediated stomatal closure as double mutants show enhanced ABA sensitivity and drought tolerance [86]. Metabolites like trehalose also affect stomatal movement. For example, the overexpression of *AtTRE1* gene encoding trehalase shows sensitivity towards the ABA-dependent stomatal closure [87].

### 2.2. Seed Dormancy

**Figure 2:**
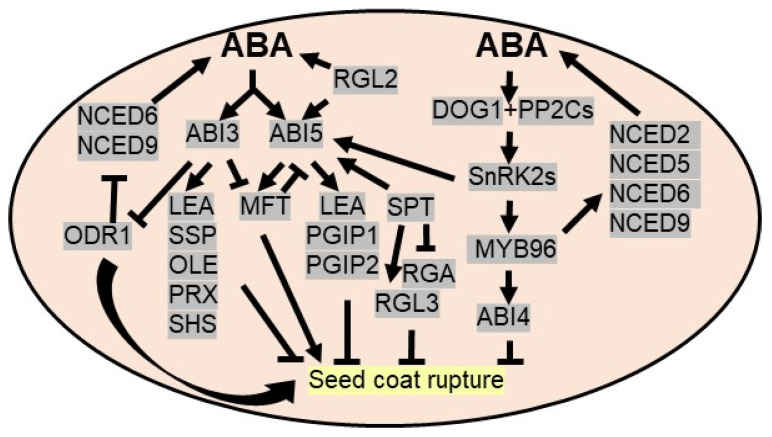
**ABA triggers seed dormancy to avoid drought stress.** ABI3 (abscisic acid insensitive 3), a major downstream component of ABA signaling, triggers *SSP* (seed storage protein), OLE (Oleosin), *LEA* (late embryogenesis abundant), *PRX* (peroxiredoxin-like proteins) and *SHS* (small heat shock proteins) to promote seed dormancy. DELLA protein RGA-LIKE2 (RGL2), a negative regulator of GA signaling, induces ABI5 TFs to upregulate *LEA* and *PGIP1*/*PGIP2* (POLYGALACTURONASE INHIBITING PROTEIN) genes and triggers endogenous ABA concentration to promote seed dormancy. ABI3 downregulates *ODR1* (reversal of reduced dormancy5 1 or *RDO51*) as it inhibits ABA biosynthesis enzymes, *NCED6* (9-cis-epoxycarotenoid dioxygenase6) and *NCED9*. ABI3 and ABI5 regulate *MFT* (MOTHER OF FT AND TFL1) expression and *MFT* via negative feedback regulation represses *ABI5*. DOG1 (delay of germination-1), a master regulator of dormancy, is induced by ABA/drought. DOG1 binds to PP2Cs to derepress ABA signaling by SnRK2. MYB96 TF activates ABA biosynthesis enzymes to increase the endogenous ABA concentration and induces *ABI4* to inhibit seed germination. SPT (SPATULA) regulates the expression of *ABI5*, *RGA* and *RGL3* to promote seed dormancy.

Seed dormancy, a temporary quiescent state, is an adaptive mechanism to prevent viable seed germination under unfavorable growth conditions. To avoid the harsh and challenging growing season or drought, seeds in the dormant stage suppress metabolic processes and eventually germinate under favorable conditions. A seed can maintain its viability at a dormant stage for long duration [88], widely dictated by environmental factors and plant hormones. A dynamic balance between abscisic acid (ABA) and gibberellins (GAs) regulates seed dormancy and germination. ABA promotes and maintains seed dormancy, whereas GAs break seed dormancy. The levels of ABA upsurge during embryonic development and remains high in mature seed. ABA inhibits the cell wall loosening of embryos and prevents water imbibition required for germination. Several genetic analyses demonstrated the involvement of ABA-mediated signaling in seed dormancy. For example, abscisic acid insensitive 3 (ABI3), a major downstream component of ABA signaling, is a pivotal regulator of seed dormancy and desiccation tolerance during embryogenesis by affecting ABA signaling and ABA biosynthesis. ABI3 activates the expression of ABA-inducible genes, like seed storage proteins (*SSPs*), oleosin (*OLE*), late embryogenesis abundant (*LEA*) proteins, peroxiredoxin-like proteins (*PRXs*), and small heat shock proteins (*SHSs*) [89]. In addition, ABI3 transcriptionally represses *ODR1* (reversal of reduced dormancy5 1 or RDO51) by binding to its promoter at the proximal RY motif. ODR1 also interacts with bHLH57 to negatively regulate the expression of ABA biosynthetic genes, like 9-cis-epoxycarotenoid dioxygenase6 (*NCED6*) and *NCED9*, required for seed dormancy [90,91]. ABI5, another major downstream component of ABA signaling, is activated by SnRK2s via phosphorylation before binding to various promoters consisting ABRE/G-box elements like *LEA* genes [92,93]. ABI5 also interacts physically with ABI3 to synergistically regulate promoters of many ABA-induced genes [94]. Predominantly, ABI5 inhibits embryo development by stimulating a group of *LEA* genes to combat drought stress. ABI5 also induces *PGIP (POLYGALACTURONASE INHIBITING PROTEIN)* and *PGIP2* encoding polygalacturonase inhibitors to inhibit seed germination in Arabidopsis [95] (Figure 2).

DELLA proteins are a GRAS family of transcription factors and serve as a convergence point of ABA and GA signaling pathways. DELLA inhibits the GA responses concomitantly, activating ABA to stimulate seed dormancy to escape from the water deficit condition. In Arabidopsis, out of five DELLA proteins (GA INSENSITIVE (GAI), REPRESSOR OF GA1-3 (RGA), RGA-LIKE1 (RGL1), RGL2 and RGL3), RGL2 acts as a major repressor of seed germination as it induces ABI5 expression as well as endogenous ABA concentration when GA levels are low. Similar to ABI5, the expression of *RGL2* is stimulated by exogenous ABA [92]. The expression of *MFT* (*MOTHER OF FT AND TFL1*), a phosphatidylethanolamine-binding protein, is positively and negatively regulated by ABI5 and ABI3, respectively, to deploy ABA signaling pathways by suppressing ABI5 during seed germination via a negative feedback regulation [96]. Another TF, SPATULA (SPT), acts as both dormancy promoter and repressor by regulating the expression of *ABI5*, *RGA* and *RGL3* in Arabidopsis [97,98]. One of the R2R3 TFs, MYB96, transcriptionally regulates ABA biosynthetic genes like *NCED2*, *NCED5*, *NCED6,* and *NCED9* as well as GA biosynthetic genes like *GA3ox1* and *GA20ox1* to sustain a balance between ABA and GA, thereby regulating seed dormancy [99]. Catabolism of embryonic lipid reserves (triacylglycerol) assists the seed germination event via acting as energy source. ABI4, another positive regulator of ABA signaling, which is essential for inhibiting seed germination by lipid breakdown, is controlled by MYB96 [100]. In addition, WRKY2 TF acts as a negative regulator of ABA-mediated seed dormancy in Arabidopsis [101] (Figure 2).

Apart from these, a heme-binding protein, i.e., delay of germination-1 (DOG1), acts as a crucial regulator of dormancy as it binds to PP2Cs (ABA-hypersensitive germination 1/AHG1, AHG2) to positively regulate ABA signaling. Thus, *dog1* mutant seeds display a non-dormancy phenotype [102] (Figure 2). Under abiotic stresses like drought, bZIP67 TF, epigenetic regulation and alternative splicing regulate the expression levels of DOG1 to control seed dormancy [103]. Homologues of DOG1 in wheat (TaDOG1L4) promote seed dormancy as confirmed by overexpression and RNA interference studies [104]. Like Arabidopsis, TaDOG1L4 interacts with TaPP2C-a10 to modulate the ABA signaling mechanism in wheat seed dormancy [105].

### 2.3. Cuticular Wax Biosynthesis

The plant cuticle is an extracellular, thick, waxy layer that remains outside part of the epidermis to protect against a dehydrating environment, UV radiation, pathogen entry, and other abiotic stresses. The primary constituent of the plant cuticle is a macromolecular scaffold of cutin and waxes. These waxes are organic solvent-soluble lipids, typically derived from very-long-chain fatty acids (C20–C34) [106]. During the transition from an aquatic to a land lifestyle, plants were exposed to a set of challenges in the terrestrial environment, including drought, high temperature, exposure to UV radiation etc. In order to sustain under such a challenging environment, plants would have necessitated some morphological and physiological features. Establishing a hydrophobic surface layer or cuticle was one of the adaptive milestones to retain water inside plant cells under dehydrating conditions [107]. Apart from reducing leaf transpiration and maintaining stomatal conductance, cuticular wax can act as a photoprotective layer of PS II complex under drought stress in wheat [108].

**Figure 3:**
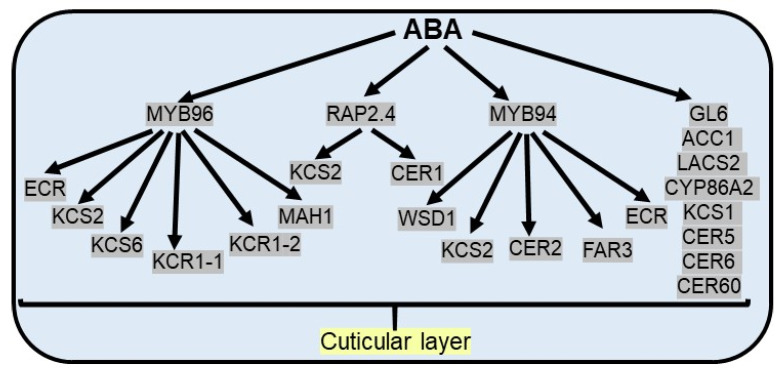
**ABA promotes the cuticular wax accumulation under drought stress.** ABA responsive MYB96 TF activates cuticular wax biosynthesis by upregulation of *ECR* (*trans-2-enoyl-CoA reductases*), *KCS2/6* (*3-ketoacyl-CoA synthetases*), *KCR1-1/1-2* (*3-ketoacyl-CoA reductases*) and *MAH1* (*mid-chain alkane hydroxylase 1*) genes. ABA responsive MYB94 TF activates cuticular wax biosynthesis by upregulation of *WSD1* (*wax synthase/acyl-CoA: diacylglycerol acyltransferase*), *KCS2*, *CER2* (*eceriferums*), *FAR3* (*fatty acyl-CoA reductase*) and *ECR* genes. Apart from MYB TF, ABA responsive RAP2.4 TF activates cuticular wax biosynthesis by upregulation of *KCS2* and *CER1*. In addition, several cuticular wax biosynthetic genes and cuticle-associated genes [*GL6* (*glossy* gene), *ACC1* (*acetyl-CoA carboxylase 1*), *LACS2* (*long-chain acyl-CoA synthetase 2*), *KCS1*, *CYP86A2* (*cytochrome P450*), *CER5*, *CER6*, *CER60*] regulate ABA induced cuticular wax biosynthesis.

Cuticular wax composition can vary considerably within the same plant during drought conditions, viz. an increased percentage of alkane in total wax is observed under water deficit conditions [107,109,110]. In addition, wax load per unit area and cuticle thickness can substantially increase in a dehydrating environment. The *wax ester synthase* (*WSD1*) gene is upregulated in water deficit conditions, resulting in an increased cuticular wax load in leaves and stems of Arabidopsis [111]. Similarly, up-regulation of some genes in the aliphatic wax biosynthetic pathway enhances cuticular wax load, including wax esters in grape berries under drought [112]. Altering cuticular wax accumulation by intracellular trafficking and augmented expression of candidate genes in the fatty acid biosynthesis pathway is regulated by the *glossy* gene (*GL6*), causing slower water losses to survive in water deficit conditions [113] (Figure 3).

ABA has been established as an important regulator, which leads to the increase in cuticular wax biosynthetic genes and cuticle-associated genes, including *acetyl-CoA carboxylase 1* (*ACC1*), *long-chain acyl-CoA synthetase 2* (*LACS2*), *3-ketoacyl-CoA synthase* (*KCS1*), *cytochrome P450* (*CYP86A2*), and *eceriferums* (*CER1*, *CER2*, *CER5*, *CER6*, *CER60*) [107,114]. In Arabidopsis, ABA treatment induces the expression of the *CER6*, which causes an increase in surface wax accumulation in Arabidopsis [115]. *BnKCS1-1*, *BnKCS1-2*, and *BnCER1-2* promote cuticular wax production in *Brassica napus* and thereby increase resistance to water deficit conditions [116]. Apart from stomatal closure and the regulation of seed dormancy, ABA-responsive MYB96 TF plays a substantial role in the biosynthesis of cuticular wax by binding with the promoter of fatty acid elongating enzymes like *3-ketoacyl-CoA synthetases* (*KCS*), *3-ketoacyl-CoA reductases* (*KCR*), *3-hydroxyacyl-CoA dehydratases,* and *trans-2-enoyl-CoA reductases* (*ECR*), essential for cuticular wax biosynthesis. The same is validated from the contrasting phenotypes observed, like enhanced levels of epicuticular wax crystals on the leaf surface in *MYB96* overexpression lines and reduced levels of cuticular wax in *myb96-1* mutants [54]. The expression of multiple wax biosynthetic genes, like *KCS2*, *KCS6*, *KCR1-1*, *KCR1-2*, *ECR,* and *MAH1* (*mid-chain alkane hydroxylase 1*), is significantly enhanced as a result of the overexpression of *MYB96* in *Camelina sativa* [117]. Under drought stress conditions, another R2R3 TF MYB94 along with MYB96 additively upregulate the expression of wax biosynthetic genes to prevent the loss of water from aerial organs as double mutants (*myb96myb94*) show an additional reduction in wax load and transcript level of wax biosynthetic genes than single mutants [118]. MYB94 regulates wax biosynthesis genes via direct binding to the promoter of the *WSD1* (*wax synthase/acyl-CoA: diacylglycerol acyltransferase*), *KCS2/DAISY* (*β-ketoacyl-CoA synthase*), *CER2*, *FAR3* (*fatty acyl-CoA reductase*), and *ECR* (*enoyl-CoA reductase*) genes in Arabidopsis [119]. In maize, ZmFDL1/MYB94 has been reported to function as a positive regulator in cuticular wax biosynthesis under drought conditions [120]. Apart from MYB TFs, AP2/ERF TF family members activate wax biosynthetic genes to enhance drought resistance. For example, ABA and drought inducible RAP2.4 TF increase cuticular wax biosynthesis via direct interaction with CCGAC or GCC consensus motifs in promoters of *KCS2* and *CER1* genes in Arabidopsis [121] (Figure 3).

### 2.4. Leaf Senescence

Organ senescence causes programmed cell death regulating the development of all living organisms. Leaf senescence in plants is not only age-related, but also acts as the long-term adaptive mechanism under drought conditions facilitating minimal water loss for survival and completion of their life cycle. Leaf senescence and abscission, involving the termination of photosynthesis, increase of reactive oxygen species (ROS), accumulation of exhausted materials to dying cells, and remobilization of nutrients from senescent leaves to young leaves, meristem, or storage organs, is predominantly regulated by various factors modulated by various phytohormones, viz. ABA, ethylene, jasmonic acid, salicylic acid, and strigolactones [122,123,124]. Among these, ABA is a critical phytohormone that mediates leaf senescence. Accumulation of ABA by overexpression of *OsNCED5* accelerates senescence in transgenic rice and contrasting phenotype has been detected in *nced5* mutant [125]. ABA is involved in the biosynthesis of ethylene by inducing 1-aminocyclopropane-1-carboxylic acid (ACC) synthase to promote senescence as ethylene is reported to induce organized cell disassembly and nutrient mobilization from senescent leaves to young organs [126,127].

**Figure 4:**
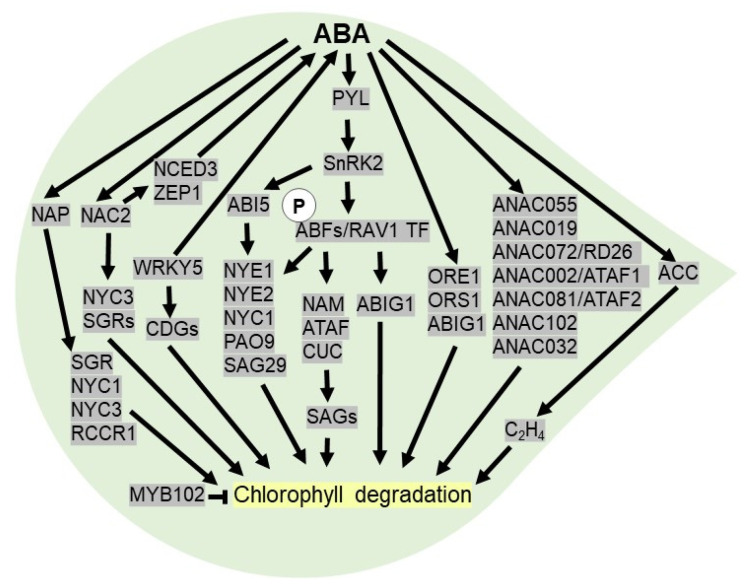
**ABA activates leaf senescence under drought stress.** After perception of ABA, PYL receptor triggers SnRK2 to phosphorylates ABFs (ABA-responsive element-binding factors) and RAV1 (ABA-insensitive 3/VP1) TF, which binds to promoter of *NAC* (*NAM*, *ATAF* and *CUC*) TFs to regulate downstream *SAGs* (senescence-associated genes) genes required for chlorophyll degradation. ABFs and ABI5 induce *NYE1* (*non-yellowing 1*), *NYE2*, *NYC1* (*Non-Yellow Coloring1*) and *PAO9* (*pheophorbiden a oxygenase*) to induce leaf senescence. ABA inducible NAC TFs (NAP, ORE1, ORS1, ANAC055, ANAC019, ANAC072/RD26, ANAC002/ATAF1, ANAC081/ATAF2, ANAC102 and ANAC032) accelerate chlorophyll degradation. ABA promotes leaf senescence by stimulating ACC (1-aminocyclopropane-1-carboxylic acid) synthase essential for ethylene biosynthesis. In rice, WRKY TF modulates leaf senescence by triggering ABA biosynthetic genes and *CDGs* (chlorophyll degradation genes). ABA inducible NAP TF triggers *SGR* (*STAY-GREEN*), *NYC1*, *NYC3* and *RCCR1* *(red chlorophyll catabolite reductase 1)* to promote chlorophyll degradation. ABA inducible NAC2 TF triggers *NYC3*, *SGRs* and ABA biosynthesis by activating *NCED3* and *ZEP1* (*zeaxanthin oxidase*) to promote chlorophyll degradation.

In ethylene-independent manner, core ABA signaling components like PYL receptors, PP2Cs phosphatase and protein kinases like SnRK2 play a crucial role in regulating leaf senescence. For example, the overexpression of *PYL9* under stress-inducible promoter in Arabidopsis increases ABA sensitivity and drought resistance by promoting leaf senescence, thereby facilitating water transport to developing tissues [128]. Accumulation of ABA under drought conditions activates SnRK2 mediated phosphorylation of ABFs (ABA-responsive element-binding factors) and RAV1 (ABA-Insensitive 3/VP1) TFs via ABA signaling. The phosphorylated ABFs and RAV1 bind to ABRE motif elements in the promoter of *NAC* (*NAM*, *ATAF,* and *CUC*) TFs, which are likely to act as crucial regulators in mediating ABA-triggered leaf senescence by modulating downstream *SAGs* (senescence-associated genes). Precocious leaf senescence has been observed in Arabidopsis after overexpression of ABA inducible *NAC* TFs, like *NAP*, *ORESARA1* (*ORE1*), and *Oresara 1 sister 1* (*ORS1*) [128,129,130]. In Arabidopsis, an ABA-inducible group of stress-responsive NAC TFs, SNAC-As, including *ANAC055*, *ANAC019*, *ANAC072/RD26*, *ANAC002/ATAF1*, *ANAC081/ATAF2*, *ANAC102,* and *ANAC032*, triggers leaf senescence by activating a set of ABA-inducible genes independent of AREB/ABFs [131]. In rice, OsNAC2 plays a fundamental role in leaf senescence as it transcriptionally activates *OsNYC3* (non-yellow coloring1) and *OsSGR* (*STAY-GREEN*) genes (Figure 4). Additionally, OsNAC2 modulates ABA biosynthetic (*OsNCED3* and *OsZEP1*) and catabolic genes (*OsABA8ox1*) to increase ABA levels. Thus, leaf senescence is significantly delayed in *OsNAC2-RNAi* lines, whereas the overexpression of *OsNAC2* accelerates senescence in transgenic rice plants [132].

To date, there are pieces of evidence indicating that chlorophyll breakdown is a marker of leaf senescence and ABA can promote the degradation of chlorophyll under drought [133]. The ABA-responsive element (ABRE)-binding TFs, viz ABI5 or EEL (ENHANCED EM LEVEL), ABF2, ABF3, and ABF4, which are activated by PYLs-PP2C-SnRK2 core-sensing system, trigger *NYE1* (*NON-YELLOWING 1*) or *SGR1*, *NYE2*, *NYC1,* and *PAO9* (*pheophorbiden a oxygenase*) to accelerate chlorophyll degradation. In addition, ABF can directly activate the *SAG* gene, like *SAG29* essential for leaf senescence [134]. In rice, OsNAP is a positive regulator of early leaf senescence as it induces chlorophyll degradation genes (*CDGs*), such as *SGR*, *NYC1*, *NYC3,* and *RCCR1 (red chlorophyll catabolite reductase 1)* [135] (Figure 4). A WRKY TF, OsWRKY5, promotes ABA biosynthesis and chlorophyll degradation genes, leading to early leaf senescence [136]. In addition, the transcript levels of *ABIG1* (ABA insensitive growth 1) or *HAT22* (homeobox from *Arabidopsis thaliana* 22) is increased in the presence of ABA. In Arabidopsis, ABIG1, a part of an ABA signaling pathway, accelerates leaf senescence by activating multiple pathways in drought conditions [137]. On the other hand, OsMYB102 delays the leaf senescence in rice as it acts as a negative regulator of ABA accumulation and signaling [138]. Similarly, a WRKY TF in cotton, GhWRKY91, acts as a negative regulator of ABA- and drought-induced leaf senescence [139].

### 2.5. Root and Shoot Length

Roots are essential for plant growth and development as they utilize soil resources via the uptake of water and nutrients. Under water-limited conditions, plant sustainability, as well as productivity, depends on root traits like root length, root diameter, root angle, root density, lateral root number, root hair density etc. To support existing shoots in water deficit condition, plants produce considerably longer roots with decreased diameter, which is vital to acquire the available water at depths in the soil and thus, maintenance of root elongation at low water potentials (ψ_w_) is an essential adaptive feature under dry conditions [140]. Typically, drought stress activates ABA to accumulate in the roots, and enhanced drought duration increases the level of ABA in the root apex to trigger the adaptive morphological changes, including root tip swelling and root apical meristem premature (RAM) differentiation [141,142]. Accumulation of ABA also regulates the architecture of the root system and hydraulic conductivity or unit length root conductance [143,144]. Predominantly, root water uptake in plants is influenced by ABA inducible water channel proteins named aquaporins that alter cell water permeability to maintain cellular water and osmotic homeostasis [145,146].

**Figure 5:**
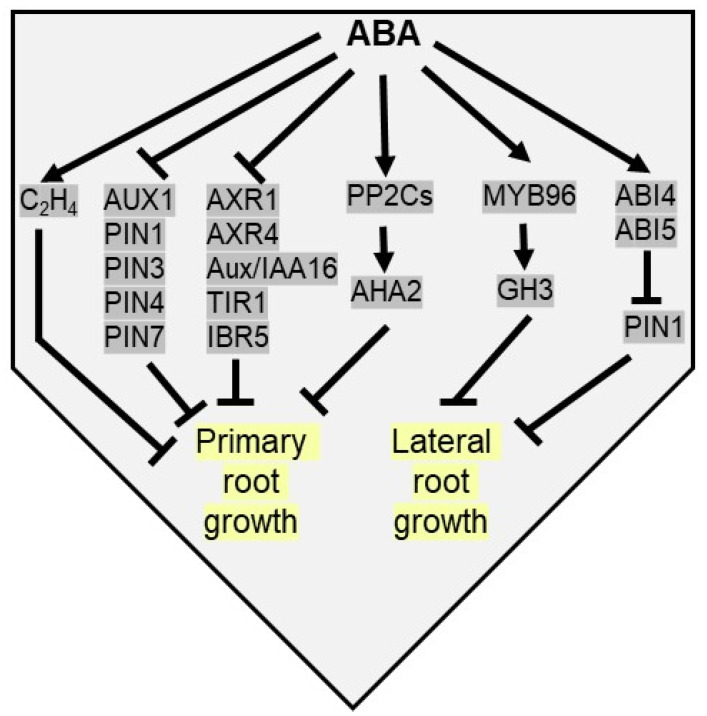
**ABA inhibits both primary and lateral root elongation under drought stress.** ABA activates ethylene biosynthesis to inhibit primary root growth. ABA inhibits auxin transport [*AUX1, PIN1* (*pin-formed 1*)*, PIN3, PIN4* and *PIN7*) and auxin signaling genes [*AXR1* (*auxin resistant 1*)*, AXR4, Aux/IAA16*, *TIR1* (transport inhibitor response 1) and *IBR5* (IBA response 5)] to arrest primary root growth. ABA upregulates PP2Cs to dephosphorylate AHA2 (H^+^-dependent adenosine triphosphatase 2) to inhibit primary root growth. ABA inducible MYB96 TF upregulates *GH3* (gretchen hagen 3) to inhibit lateral root elongation. ABA restricts lateral root growth by suppressing PIN1 proteins via ABI4 and ABI5.

The underlying mechanisms that allow morpho-physiological effects on root growth by ABA are intricate as they connect with diverse hormonal regulatory networks. For example, in ABA-deficient seedlings, increased ethylene accumulation inhibits root growth, and therefore ABA maintains root growth under low water conditions by inhibiting ethylene production [147]. To promote root stem cell maintenance, a low concentration of ABA inhibits quiescent centre (QC) division and differentiation of stem cells and their daughter cells in primary root meristem [148,149]. Typically, moderate water stress or low concentration of ABA positively modulates root growth by manipulating auxin levels via auxin transport and auxin signaling [150]. Root-specific NF-Yb21 (NUCLEAR FACTOR-Y) TF interacts with FUS3 (FUSCA3) to promote ABA biosynthesis via activation of *NCED3*, which in turn promotes auxin transport leading to root growth and drought tolerance in populus [151]. On the other hand, high concentrations of ABA inhibit root growth by reducing the expression of auxin transport genes in Arabidopsis roots, viz. *AUX1*, *PIN1 (PIN-FORMED 1)*, *PIN3*, *PIN4*, and *PIN7* [152,153]. As a result, decreased sensitivity to ABA is detected in *aux1* (*auxin resistant 1*), *axr4,* and *pin2* mutants. Apart from auxin transport genes, several signaling components of auxin like *AXR1* (Auxin resistant 1), *AXR4*, *Aux/IAA16* (Aux/Indole-3-acetic acid), *TIR1* (transport inhibitor response 1), and *IBR5* (IBA response 5) are downregulated by ABA, resulting in the suppression of primary root growth [154]. Apart from these, a recent study depicts a model of ABA concentration-dependent root growth modulation by H^+^ extrusion across the plasma membrane. High ABA concentration upregulates PP2Cs, which dephosphorylate Thr^947^ of H^+^-dependent adenosine triphosphatase 2 (AHA2) after binding its C-terminal R domain, resulting in primary root growth arrest of Arabidopsis by inhibition of apoplastic H^+^ efflux. Whereas, low ABA concentration positively regulates root growth by derepressing AHA2 and H^+^ extrusion via ABA receptor-mediated inhibition of PP2C activity [155] (Figure 5).

The inhibition of lateral root (LR) growth is another adaptive phenotypic response of plants against drought stress. Plants adapt to drought by restricting the horizontal spread of lateral growth and utilizing energy in primary root elongation to acquire water in water deficit soil [156]. Thus, despite a similar number of lateral root primordia in both wild type and ABA related mutants in Arabidopsis, root primordia fail to elongate in mutants [156,157]. Predominantly, lateral root initiation is promoted by auxin-dependent cell cycle-related genes. In Arabidopsis, ABA-induced MYB96 TF enhances the expression of *GH3*, encoding an auxin-amido synthetase, which inhibits lateral root elongation by inactivating endogenous auxin pool [80]. An auxin efflux carrier gene *PIN1* modulates polar auxin transport from the shoot to root apices, affecting lateral root development. ABA negatively regulates *PIN1* expression by its downstream signaling components, viz ABI4 and ABI5 (Figure 5). Thus, LR initiation and the elongation of emerged LRs are inhibited in ABI4 overexpression lines of Arabidopsis [154,158,159,160].

Although different sensitivity is observed between shoot and root tissues under water-limited conditions, drought-induced ABA typically inhibits shoot growth. Water deficit and ABA cause prevention of shoot growth of maize, but it is derepressed in fluridone-treated seedlings as it targets carotenoid biosynthetic pathway to reduce endogenous ABA pool [161]. Similarly, ABA accumulation under low water potential has been reported to prevent the shoot growth of soybean [162]. Predominantly, endogenous ABA restricts ethylene production under water deficit conditions to maintain shoot growth in the early and late developmental stages [143,163,164]. Apart from that, it has been reported that OsbZIP23 TF plays a key role in conferring drought tolerance by enhancing the expression of many stress related genes, like *LEA*, *RD22,* etc., in rice [165].

## 3. Conclusions and Future Perspectives

**Figure 6:**
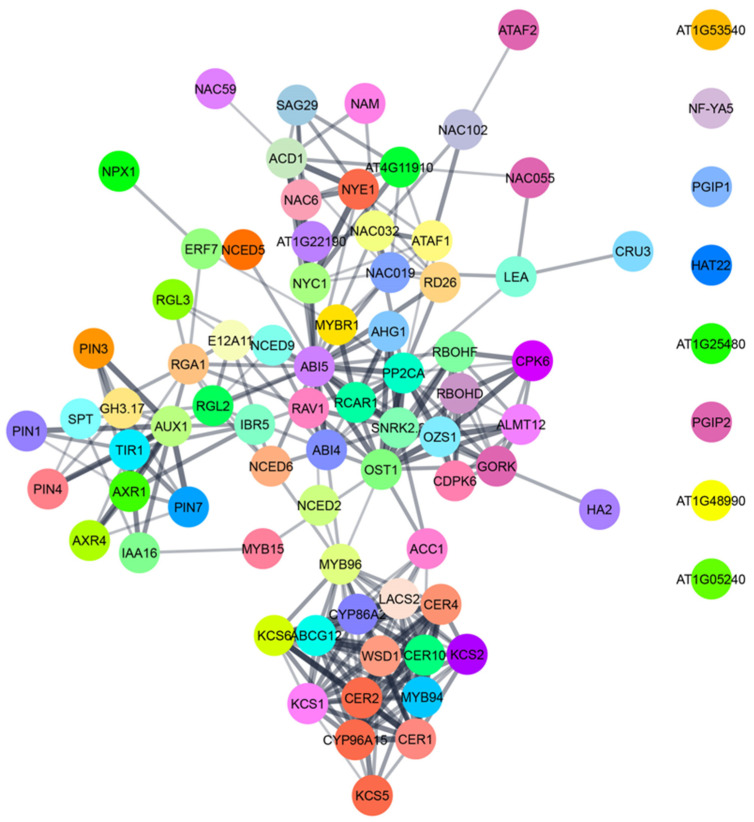
A network of proteins involved in ABA-mediated physiological adaptations like stomatal closure, seed dormancy, cuticular wax biosynthesis, leaf senescence and alteration of root and shoot growth in *Arabidopsis thaliana*. The data for AGI locus code was collected from TAIR—(arabidopsis.org (accessed on 16 November 2021)). The interaction network was prepared by using STRING (string-db.org (accessed on 16 November 2021)) and represented by using Cytoscape software.

Plants are exposed to a changing environment throughout their existence. Drought is one of the most prevalent global problems that negatively affect agricultural production, reducing net photosynthesis by altering plant carbon allocation and metabolism. To cope with drought, plants can elicit physiological and biochemical responses aimed at enhancing resistance. Phenotypic plasticity, including modifications of vegetative and reproductive architecture, is essential for resistance to water deficiency. The developmental plasticity of the plant organs is regulated by endogenous ABA level, which precisely regulates numerous signaling proteins, TFs, and even ABA biosynthetic genes. Extensive cross-talk among those proteins leads to the formation of complex signaling networks (Figure 6). Here, we provided considerable phenotypic and genetic evidence for the ABA-mediated drought stress resistance in plants through various molecular mechanisms, which is critically important to understanding the fundamental biology that underscores the stress resistance phenotype. This detailed knowledge about how plants modify when challenged by drought is essential to enhance drought-stress resistance in different crop plants.

Drought stress responses are coordinated by complex signaling networks. It is implausible that a single gene alone regulates plant drought tolerance. QTL mapping facilitated the establishment of relationships between drought stress tolerance and agronomic and physiological traits by identifying genomic regions linked with traits of interest. Recent genomics tools, molecular techniques, and precise phenotype analysis detected several candidate genes for crop drought tolerance. Multiple cross-talk among different regulatory networks during drought stress makes it a challenge to discriminate those interactions that most effect under water deficit conditions. Therefore, it is critically important to recognize convergent points in the drought stress response circuitry essential for translational research. In future, a systems biology approach, including transcriptomics, proteomics, and metabolomics, high-throughput phenotyping, functional characterization of novel regulatory candidate genes, and detailed study of epigenetics, will be needed to precisely manipulate physiological processes for developing drought-tolerant crops.

## Data Availability

Not applicable.
